# 

**Published:** 1888-06-09

**Authors:** 


[THIRTY-TWO PAGES, INCLUSIVE OF FOUR EXTRA.]

PRICE ONE PENNY.

No. 89, Vol. IV.—June 9th, 1888.

[Registered at the General Post Office as a Newspaper.]

Per Annum, 6s. 6d., post free.
Monthly Parts, 6d.; post free, 7½d.
Ditto per Ann., 7s. 6d. post free.

